# Mask-Guided and Fidelity-Constrained Deep Learning Model for Accurate Translation of Brain CT Images to Diffusion MRI Images in Acute Stroke Patients

**DOI:** 10.1007/s10278-025-01649-6

**Published:** 2025-09-02

**Authors:** Muhammad Adil Khalil, Mariusz Bajger, Anthony Skeats, Chris Delnooz, Andrew Dwyer, Gobert Lee

**Affiliations:** 1 College of Science and Engineering, Flinders University, Bedford Park, Adelaide, SA 5042 Australia; 2 Micro-X Ltd, Tonsley, Adelaide, SA 5042 Australia; 3 South Australian Health and Medical Research Institute (SAHMRI), North Terrace, Adelaide, SA 5000 Australia

**Keywords:** Deep learning, CT/MRI translation, Computed tomography, Magnetic resonance imaging, Stroke diagnosis

## Abstract

The early and precise diagnosis of stroke plays an important role in its treatment planning. Computed Tomography (CT) is utilised as a first diagnostic tool for quick diagnosis and to rule out haemorrhage. Diffusion Magnetic Resonance Imaging (MRI) provides superior sensitivity in comparison to CT for detecting early acute ischaemia and small lesions. However, the long scan time and limited availability of MRI make it not feasible for emergency settings. To deal with this problem, this study presents a brain mask-guided and fidelity-constrained cycle-consistent generative adversarial network for translating CT images into diffusion MRI images for stroke diagnosis. A brain mask is concatenated with the input CT image and given as input to the generator to encourage more focus on the critical foreground areas. A fidelity-constrained loss is utilised to preserve details for better translation results. A publicly available dataset, A Paired CT-MRI Dataset for Ischemic Stroke Segmentation (APIS) is utilised to train and test the models. The proposed method yields MSE 197.45 [95% CI: 180.80, 214.10], PSNR 25.50 [95% CI: 25.10, 25.92], and SSIM 88.50 [95% CI: 87.50, 89.50] on a testing set. The proposed method significantly improves techniques based on UNet, cycle-consistent generative adversarial networks (CycleGAN) and Attention generative adversarial networks (GAN). Furthermore, an ablation study was performed, which demonstrates the effectiveness of incorporating fidelity-constrained loss and brain mask information as a soft guide in translating CT images into diffusion MRI images. The experimental results demonstrate that the proposed approach has the potential to support faster and precise diagnosis of stroke.

## Introduction

Stroke is one of the leading causes of death and long-term disability worldwide. Rapid imaging plays an important role in the diagnosis and management of stroke patients. It enables clinicians to differentiate between ischaemic and haemorrhagic strokes, assess the infarct size and determine the viability of the brain tissues. Computed Tomography (CT) scans are usually the first choice for stroke diagnosis because they are readily available, quick, economical and ideally suited to rule out haemorrhage. However, non-contrast CT (NCCT) has limitations in visualising early ischaemic changes, and infarcts may not be detected. MRI is more accurate in identifying stroke lesions, particularly diffusion-weighted imaging (DWI) and corresponding apparent diffusion coefficient (ADC) maps [1–4]. ADC maps derived from diffusion data quantify the tissue signal loss at increasing strengths of diffusion-sensitising gradients, with low ADC values in stroke reflecting cell wall damage and intracellular swelling, the earliest imaging manifestation of the pathology that also causes low-density oedema to be visible on CT. However, MRI is expensive and inaccessible, especially in remote areas. Furthermore, the image acquisition time of MRI is quite long, which is not optimal for stroke patients, where rapid decisions are necessary.

CT and MRI can provide complementary information [5–8]. However, the acquisition of image data from both CT and MRI will result in an increase in diagnostic time, cost and can be disruptive to healthcare logistics. To deal with this, deep learning-based methods can be utilised to generate synthetic MRI images from CT images. The synthetic MRI images would offer clinicians additional MRI-equivalent information from more available CT imaging. Over the recent years, deep learning-based models have been widely utilised for several applications in the field of medical imaging [9–15]. For the paired image-to-image translation task, Pix2Pix [16] is a widely utilised deep learning framework employing a conditional generative adversarial network (GAN) with a UNet-based generator and a patch-based discriminator for the accurate translation results. Pix2Pix employs paired data to enhance pixel-wise accuracy by using adversarial learning and *L*1 losses. Similarly, UNet [17], which is an encoder-decoder-based deep learning network, is also utilised for paired image-to-image translation. However, the acquisition of a large paired CT and MRI dataset is challenging. To the best of our knowledge, cycle-consistent generative adversarial network [18] (CycleGAN) and UNet have been used for generating synthetic MRI images from CT images [19–26]. Li et al. [19] performed supervised and unsupervised translation of brain CT images into MRI images. In their supervised translation, they trained CycleGAN and UNet with a paired CT and MRI dataset, while in unsupervised translation, an unpaired CT and MRI dataset was utilised to train CycleGAN. The results demonstrate that the supervised translation obtains better results than the unsupervised translation. Kalantar et al. [20] utilised three deep learning models, including UNet, UNet++ [27] and CycleGAN for the translation of pelvic CT images into MRI images. Their experimental results demonstrate that the CycleGAN produced sharper translated MRI images in comparison to UNet and UNet++, which generated smoother images. Kieselmann et al. [21] utilised CycleGAN to synthesise MRI images from CT images. They further utilised synthetic MRI images to train a UNet model to segment parotid glands. Their experimental results demonstrate that the UNet trained with synthetic MRI images obtains results that are close to the ground truth for the segmentation of parotid glands. Dong et al. [22] trained a CycleGAN to translate CT images into MRI images. The synthetic MRI images were then utilised to train a deep attention UNet model for multi-organ segmentation. They achieve more than 85% dice-coefficient score with the ground truth for the segmentation of three organs, including the bladder, prostate and rectum, from synthetic MRI images. Dai et al. [23] employed a pre-trained CycleGAN to synthesise head and neck MRI images from CT images. The extracted features collected from original CT and synthetic MRI images were used to train a regional convolutional neural network (R-CNN) [28] to segment head and neck organs at risk automatically. Their experimental results demonstrate the potential of utilising synthetic MRI images in radiotherapy to plan for the head and neck organs at risk. McNaughton et al. [24] trained seven different versions of UNet, including six 3D versions of UNets and a 2D version of UNet, along with 3D CycleGAN to generate synthetic MRI volumes from CT volumes. The synthetic MRI volumes were then used to guide CT scan registration with MRI atlases. The experimental results demonstrate that the synthetic MRI volumes can potentially guide the registration process.

For the stroke diagnosis, Ruben et al. [25] employed a conditional GAN-based deep learning framework for the translation of CT perfusion (CTP) maps into DWI MR images. In addition, a segmentation model was trained with the original CTP and the synthetic MR image for the segmentation of ischaemic lesions. The experimental results demonstrate that the model trained with the original CTP and the synthetic MRI image obtains slightly better segmentation results than the model trained only on the original CTP maps. However, CTP maps have a low signal-to-noise ratio, require a contrast agent, require multiple scans, which increase the exposure to radiation and are not easily accessible. Hu et al. [29] utilised a GAN model for the translation of CT images to Fluid-attenuated inversion recovery (FLAIR) MRI images. The translation results demonstrate structural similarity of more than 85% between the real and synthetic FLAIR MR images. In addition, the detection of ischaemic lesions by the clinician was performed on the real CT images and translated FLAIR MR images. The results demonstrate the improved sensitivity in the detection of lesions on synthetic FLAIR images in comparison to real CT images. This study takes FLAIR MR images as the gold standard over DW MR images. However, a multicentre study by Thomalla et al. [30] demonstrated the effectiveness of DW MR images over FLAIR MR images for ischaemic lesion detection. Garzón et al. [31] proposed a cycle-consistent GAN framework in which a traditional ResNet generator is replaced by multiple U-Net models that are concatenated with each other to preserve lesions in the synthetic DW MRI images. The results demonstrate high performance in the classification between normal and lesion slices. However, the proposed method is trained in an unsupervised setting (with an unpaired CT-MR dataset), which could limit the fidelity of the translated MR image in comparison to the supervised setting. In addition, the qualitative evaluation results demonstrate that clearly visible stroke lesions in the CT images also limit the ability of the model to translate early ischaemic changes present in the acute clinical settings. Feng et al. [32] incorporated radiomics with GANs to synthesise MRI from CT for acute ischaemic stroke patients. The proposed approach used radiomics to detect potential lesion candidate regions in the CT and extracted features from those potential regions, and selected the feature with the most information gain. The selected feature map was then given as input to the GAN along with the CT image. Feng et al. [32] also modified the generator architecture by using a residual module in the bottleneck of the generator for deeper feature learning. They also utilised a lesion feature similarity loss to preserve the lesions in the synthetic MRI image. The experimental results demonstrate that the proposed approach generated synthetic MRI images which closely resembled the original MRI images. Furthermore, the segmentation of ischaemic lesions from the synthetic MRI images also achieved a high Dice score of 0.869, respectively. However, the reliability of radiomic analysis can be affected by variations in imaging protocols and scanner parameters, which can lead to inconsistencies in feature extraction [33, 34]. Platscher et al. [35] addressed the problem of a limited dataset for stroke lesion segmentation. To deal with this problem, a synthetic MRI dataset was generated using Pix2Pix, SPADE and CycleGAN models. The experimental results demonstrate that Pix2Pix achieves better translation results than the SPADE and CycleGAN models. A U-Net is then trained with clinical data alone and clinical data augmented with synthetic data generated from the translation models. The results demonstrated that augmenting clinical data with synthetic images led to better segmentation performance than using clinical data alone. Platscher et al. [35] utilised MRI-derived anatomical segmentation maps to generate a synthetic MRI dataset, which may not be feasible in clinical scenarios where MRI is not accessible. Gutierrez et al. [36] employed a modified cycle-consistent GAN framework for the translation of CT images into FLAIR MR images. In the modified framework, the lesion mask is given as an additional input to the discriminator, and the gradient consistency loss also maintains the fidelity of the translated MR images. The experimental results demonstrate improved translation performance in comparison to traditional cycle-consistent GAN. Furthermore, the results also illustrate the preservation of ischaemic lesions in the synthetic FLAIR MR images. Garzón et al. [37] proposed a GAN-based deep learning framework that uses a UNet generator and a patch-based discriminator for the synthesis of FLAIR MR sequences from the non-contrast CT images. The proposed framework by Garzón et al. [37] utilised binary and dilated class weight maps to enhance the preservation of lesions in the translated images. The experimental results demonstrated that the translated FLAIR sequences closely preserve the size and location of the lesions. However, the modified framework by Gutierrez et al. [36] and Garzón et al. [37] translates CT images into FLAIR MR images instead of DW MR images, which are more effective in the detection of ischaemic lesions [38].

The models may be trained in either a supervised (paired) or unsupervised (unpaired) manner. However, research has demonstrated that models trained in a supervised manner have performed better [19, 21, 39]. The supervised models need big datasets with large quantities of paired sets, which are challenging to acquire [40–42]. For accurate translation, deep learning-based models must locate and concentrate on the critical regions in the dataset. Recently, AttentionGAN [43] has been proposed in natural image processing, which utilises attention modules to locate regions of interest dynamically. In training, as the model weights are updated, the attention masks for the same input can vary across iterations, which can result in inconsistent focus on important regions, potentially leading to suboptimal translation results. Furthermore, attention modules might not be able to extract meaningful attention patterns from the small dataset, which will result in sub-optimal translation results. AttentionGAN uses attention masks as a hard filter, which might also result in sub-optimal translation outcomes if accurate attention masks are not generated.

In this study, we present a deep learning-based method for accurately translating CT images into MRI diffusion ADC maps of stroke patients. Firstly, the pre-defined brain mask information is concatenated with the input images, allowing it to be used as a soft guide compared to hard filtering. Secondly, inspired by the study of Johnson et al. [44] in which fidelity of the natural images was preserved by calculating the difference between the features of a real image and the synthetic image obtained from a pre-trained natural images model. We utilise RadImageNet [45] deep learning model that is trained on radiological images to obtain meaningful features to preserve the fidelity of the translated images.

## Materials and Methods

### Data

In this study, we utilised the publicly available A Paired CT-MRI Dataset for Ischemic Stroke Segmentation (APIS) [46]. The APIS dataset was developed from a retrospective study to gather 96 patients’ data demonstrating indications of stroke at two medical facilities from October 2021 to September 2022, comprising NCCT scans and ADC MRI sequences for each patient. All of the data was skull-stripped using optiBET, and co-registration was done with reference to the ADC sequences using Elastix. The whole APIS dataset has been divided into a training set (60 patients’ data) and a testing set (36 patients’ data). However, only the training set consisting of 60 patients was made publicly available. In this study, the whole dataset is randomly divided into 50 patients’ data (1233 axial slices) for training, five patients’ data (122 axial slices) for validation, and five patients’ data (122 axial slices) for testing the model.

**Fig. 1:**
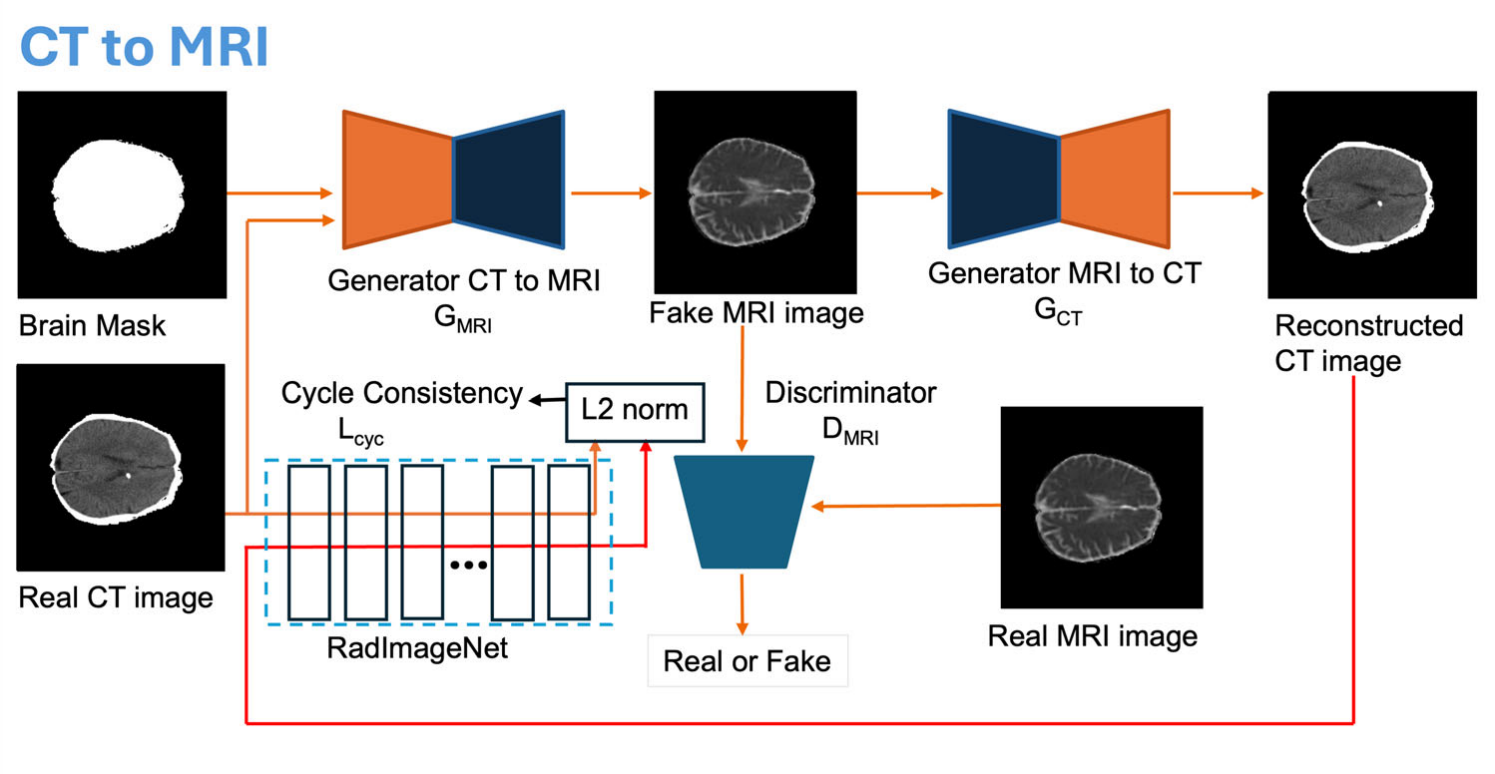
The architecture of the proposed framework

### Methods

The proposed method extends CycleGAN [18] in two ways. Firstly, the brain mask was concatenated with the input image to the CycleGAN generator. This will act as a soft guide illustrating the important regions of the model for better translation results. This study used OTSU thresholding [47] to extract the brain masks from the original CT. The brain masks were generated from the original CT images. In addition, a dilation process was subsequently applied to enhance the brain mask. Secondly, the pixel-wise cycle consistency and identity losses of the CycleGAN were replaced by the fidelity-constrained loss that maintains the consistency of the structures in the translated image. The fidelity preservation loss initially calculates the high-level feature representations of the original images and the translated images. Then, it estimates the Euclidean distance between those two feature representations to preserve structures robustly compared to pixel-based losses. To obtain the high-level feature representations, we utilise RadImageNet [45], which is trained on 1.35 million radiology images, including CT, MRI and Ultrasound images. RadImageNet captures meaningful feature representations of the original and translated images, which helps the model maintain consistency between the original and the translated images.

#### Architecture of the Proposed Network

**Fig. 2:**
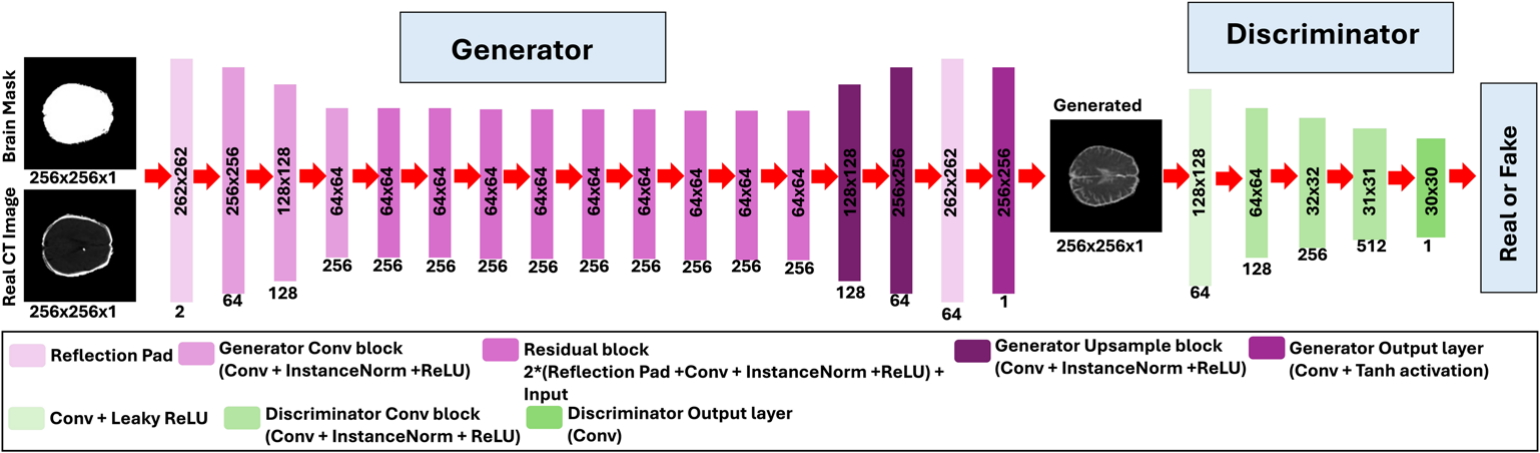
The architecture of the generator and discriminator in the proposed framework

The proposed network utilises two generator models and two discriminator models, namely $G_{CT}$, $G_{MRI}$, $D_{CT}$ and $D_{MRI}$, to develop a bidirectional mapping between CT and MRI imaging modalities. Figure 1 illustrates the detailed architecture of the proposed framework. Both the generator models have identical architecture, and the discriminator models also share the same architecture. Figure 2 presents the architecture of the generator and discriminator, respectively. The generator models are composed of an encoder and a decoder. The CT images concatenated with brain masks are given as input to the encoder, which generates meaningful features using multiple convolution and downsampling layers. The features are then provided as input to the residual blocks, which utilise multiple convolution layers and residual connections to extract the high-level features. The high-level features are then utilised by the decoder, which generates synthetic MRI images using transposed convolutions. The discriminators complement the generators to evaluate how closely the translated images resemble the original images.

#### Loss Functions

The proposed network modifies the traditional cycle consistency and identity losses of the CycleGAN [18]. Compared to the traditional CycleGAN, which relies on the L1 cycle consistency and identity losses, the proposed network employs a fidelity-constrained loss based on features extracted from the RadImageNet to evaluate cycle consistency loss and identity loss for the better preservation of the fidelity in the translated images. The adversarial loss $L_{adv}$ will be calculated as follows:

1 $$\begin{aligned} L_{G}(G_{MRI},D_{MRI}) = E[log(G_{MRI}(X_{MRI})] + E[log(1-D_{MRI}(G_{MRI}(X_{CT})] \end{aligned}$$

2 $$\begin{aligned} L_{G}(G_{CT},D_{CT}) = E[log(G_{CT}(X_{CT})] + E[log(1-D_{CT}(G_{CT}(X_{MRI})] \end{aligned}$$

3 $$\begin{aligned} L_{adv} = L_{G}(G_{MRI},D_{MRI}) + L_{G}(G_{CT},D_{CT}) \end{aligned}$$

where $X_{CT}$ and $X_{MRI}$ are the real CT and MRI images, $G_{MRI}(X_{CT})$ is the generator that takes CT images to generate synthetic MRI images, $G_{CT}(X_{MRI})$ is the generator that generates synthetic CT images with MRI images as an input while $D_{CT}$ and $D_{MRI}$ tries to evaluate the visual similarity of the synthetic CT and MRI images in comparison to real CT and MRI images. The goal is to minimise the adversarial loss function so that the generator generates synthetic images that are visually similar to the real images.

The cycle consistency loss $L_{cyc}$ helps the proposed network maintain the anatomical information by calculating the deviation between the extracted features of the translated images obtained at the end of the cycle and the original images, respectively.

4 $$\begin{aligned} L_{fid}(G_{MRI}(X_{CT}),X_{MRI}) = || \phi ( G_{MRI}(X_{CT}))-\phi (X_{MRI})||^2 \end{aligned}$$

5 $$\begin{aligned} L_{fid}(G_{CT}(X_{MRI}),X_{CT}) = || \phi ( G_{CT}(X_{MRI}))-\phi (X_{CT})||^2 \end{aligned}$$

6 $$\begin{aligned} L_{cyc} = E[L_{fid}(G_{MRI}(X_{CT}),X_{MRI})] + E[L_{fid}(G_{CT}(X_{MRI}),X_{CT}] \end{aligned}$$

where $L_{fid}$ is the fidelity preservation loss between the translated image and the original image, $\phi (.)$ is the RadImageNet feature extractor, and $||.||^{2}$ is the L2 norm, respectively

Lastly, the identity loss $L_{id}$ in the proposed network aids in maintaining the consistency between the translated images and the original images. The identity loss enforces consistency by evaluating the difference between the translated image and the original image, which is calculated as follows:

7 $$\begin{aligned} L_{fid}(G_{CT}(X_{CT}),X_{CT}) = ||\phi (G_{CT}(X_{CT}))-\phi (X_{CT})||^2 \end{aligned}$$

8 $$\begin{aligned} L_{fid}(G_{MRI}(X_{MRI}),X_{MRI}) = ||\phi (G_{MRI}(X_{MRI}))-\phi (X_{MRI})||^2 \end{aligned}$$

9 $$\begin{aligned} L_{id}= E[L_{fid}(G_{CT}(X_{CT}),X_{CT})] + E[L_{fid}(G_{MRI}(X_{MRI}),X_{MRI})] \end{aligned}$$

The total loss function of the proposed network can be expressed as follows:

10 $$\begin{aligned} L(G_{CT},G_{MRI},D_{CT},D_{MRI}) = L_{adv} + \lambda _{1}L_{cyc} + \lambda _{2}L_{id} \end{aligned}$$

**Fig. 3:**
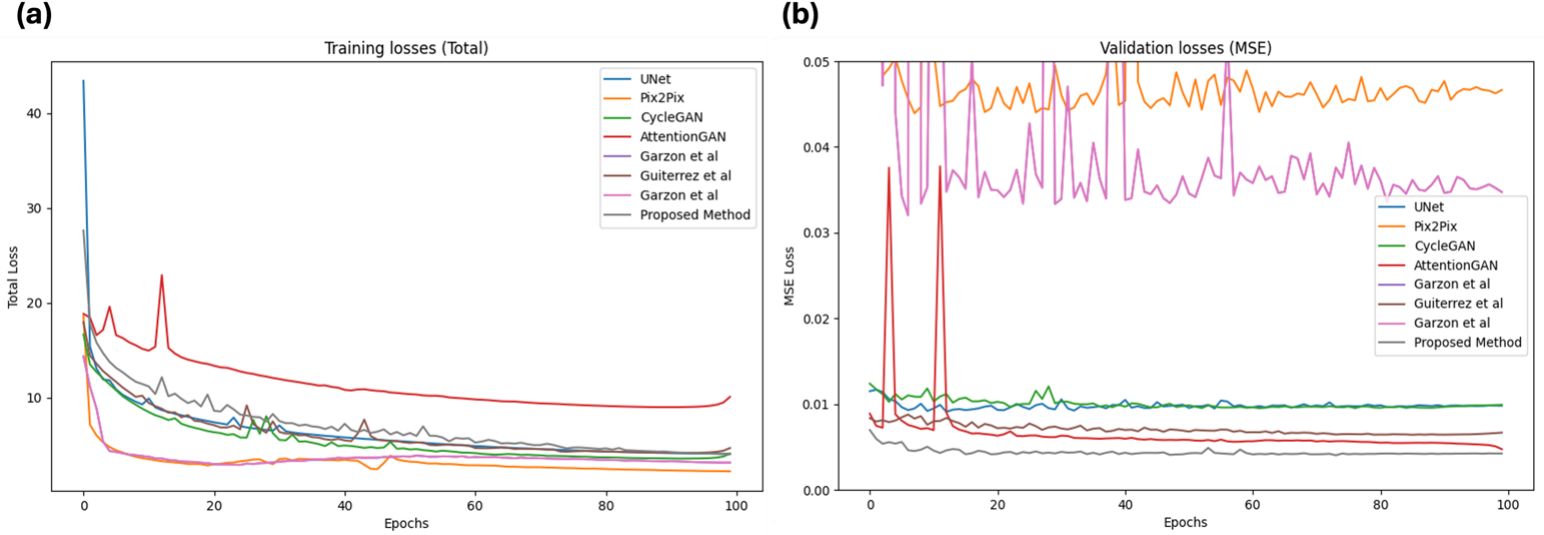
The overall training loss and MSE validation loss curves of the proposed method and the benchmark methods, including UNet [17], Pix2Pix [16], CycleGAN [18], AttentionGAN [43], Garzón et al. [31], Gutierrez et al. [36] and Garzón et al. [37]

### Experimental Settings

The proposed method was trained in a supervised manner with paired CT and MRI for 100 epochs using PyTorch on a 48GB Nvidia RTX A6000 GPU. Adam optimiser was used with a fixed learning rate of $2\times 10^{-4}$ for the first 50 epochs, then linearly reduced to zero over the last 50 epochs. The batch size of 8 was utilised to train the proposed method. For benchmarking, the proposed method is compared with four commonly used deep learning models for image translation, including UNet [17], Pix2Pix [16], CycleGAN [18] and AttentionGAN [43], and deep learning methods from three recently published studies on CT to MRI translation for stroke diagnosis, including Garzón et al. [31], Gutierrez et al. [36] and Garzón et al. [37]. All the benchmark methods were trained with the same data distribution as the proposed method. The implementation details for the benchmark method are as follows:

- UNet [17] was trained for 100 epochs with a fixed learning rate of $2\times 10^{-6}$ for the first 50 epochs, followed by linear decay to zero in the next 50 epochs. The input consisted of original CT images processed with a batch size of 8. The total loss is the combination of *L*1 and *L*2 losses. The UNet architecture consists of four encoder blocks, four decoder blocks, and a bottleneck architecture. Each encoder block consists of four convolutional layers followed by batch normalisation and ReLU activation. Each decoder block contains an upsampling layer and four convolutional layers, followed by batch normalisation and ReLU activation. The bottleneck layer featured a convolutional layer followed by batch normalisation and ReLU activation.
- Pix2Pix [16] was trained with the same training parameters as the proposed method. The original CT images were given as input. The generator and discriminator followed the original PyTorch implementation as provided in [48]. The generator loss is composed of binary cross-entropy loss and *L*1 loss.
- CycleGAN [18] was trained with a real CT image as an input with the same training parameters as the proposed method. The generator and discriminator of the CycleGAN follow the same architecture as presented in Fig. 2. The cycle consistency and identity loss consisted of a traditional *L*1 loss.
- AttentionGAN [43] was trained with original CT images as input and the same parameters as the proposed method. The architecture of AttentionGAN adapted the original PyTorch implementation as provided in [49].
- The best-performing model of Garzón et al. [31] was trained with real CT images. The generator consisted of two cascaded UNets as the generator for 100 epochs. The cascaded UNets have the same architecture as the baseline UNet [17] with the same training parameters. The cycle consistency and identity loss was consisted of traditional *L*1 loss.
- Following the original implementation of Gutierrez et al. [36], the gradient consistency loss was added to the traditional CycleGAN [18] loss. In addition, the ground truth lesion mask was given as input to the discriminator. The generator and discriminator were consistent with those presented in Fig. 2. *L*1 loss was utilised as cycle consistency and identity loss.
- The best-performing model of Garzón et al. [37] was trained by using a binary weighted lesion-aware loss. The generator and discriminator architectures followed the original PyTorch implementation of Pix2Pix2 [48]. The generator loss is the combination of *L*1 loss, binary cross-entropy loss and binary weighted lesion-aware loss.

For model evaluation, the inference weights of the best model were selected using the validation loss curves of the models. The model with the lowest validation loss after the 20th epoch was identified as the best model. Figure 3 presents the overall training and validation mean square error (MSE) loss curves of the proposed method and the benchmark methods. Using the validation loss curves, the best models of UNet [17], Pix2Pix [16], CycleGAN [18], AttentionGAN [43], Garzón et al. [31], Gutierrez et al. [36], Garzón et al. [37] and Proposed Method were selected at epochs 32, 62, 61, 99, 38, 85, 35 and 73, respectively.

**Table 1:** Quantitative evaluation results for translating CT images into ADC MRI images

Method	Number of	MSE	PSNR	SSIM (%)
	slices	(95% CI)	(95% CI)	(95% CI)
		*P* value	*P* value	*P* value
Commonly used models				
		416.86	22.11	80.21
UNet [17]	122	(388.33, 445.39)	(21.81, 22.41)	(79.09, 81.33)
		3.56$\times$ $10^{-13}$	3.56$\times$ $10^{-13}$	3.56$\times$ $10^{-13}$
		477.62	21.57	80.21
Pix2Pix [16]	22	(438.78, 516.45)	(21.23, 21.90)	(72.63, 74.47)
		3.56$\times$ $10^{-13}$	3.56$\times$ $10^{-13}$	3.56$\times$ $10^{-13}$
		434.70	21.93	79.37
CycleGAN [18]	122	(404.04, 465.37)	(21.64, 22.23)	(78.35, 80.39)
		3.97$\times$ $10^{-13}$	3.97$\times$ $10^{-13}$	3.64$\times$ $10^{-13}$
		450.28	21.81	80.81
AttentionGAN [43]	122	(416.36, 484.20)	(21.49, 22.13)	(79.80, 81.82)
		4.14$\times$ $10^{-13}$	4.14$\times$ $10^{-13}$	3.64$\times$ $10^{-13}$
Recent Literature				
		411.98	22.15	80.04
Garzón et al. [31]	122	(384.28, 439.68)	(21.86, 22.44)	(79.05, 81.03)
		3.56$\times$ $10^{-13}$	3.56$\times$ $10^{-13}$	3.56$\times$ $10^{-13}$
		432.36	22.97	79.45
Gutierrez et al. [36]	122	(400.36, 464.34)	(21.65, 22.28)	(78.42, 80.48)
		3.88$\times$ $10^{-13}$	3.88$\times$ $10^{-13}$	3.56$\times$ $10^{-13}$
		821.22	19.33	73.29
Garzón et al. [37]	122	(742.78, 899.66)	(18.91, 19.73)	(72.31, 74.28)
		3.56$\times$ $10^{-13}$	3.56$\times$ $10^{-13}$	3.56$\times$ $10^{-13}$
		**197**.**45**	**25**.**50**	**88**.**50**
Proposed Method	122	**(180.80, 214.10)**	**(25.10, 25.92)**	**(87.50, 89.50)**
		–	–	–

The best-performing model is highlighted in bold

## Results

### Comparison Benchmarking

**Fig. 4:**
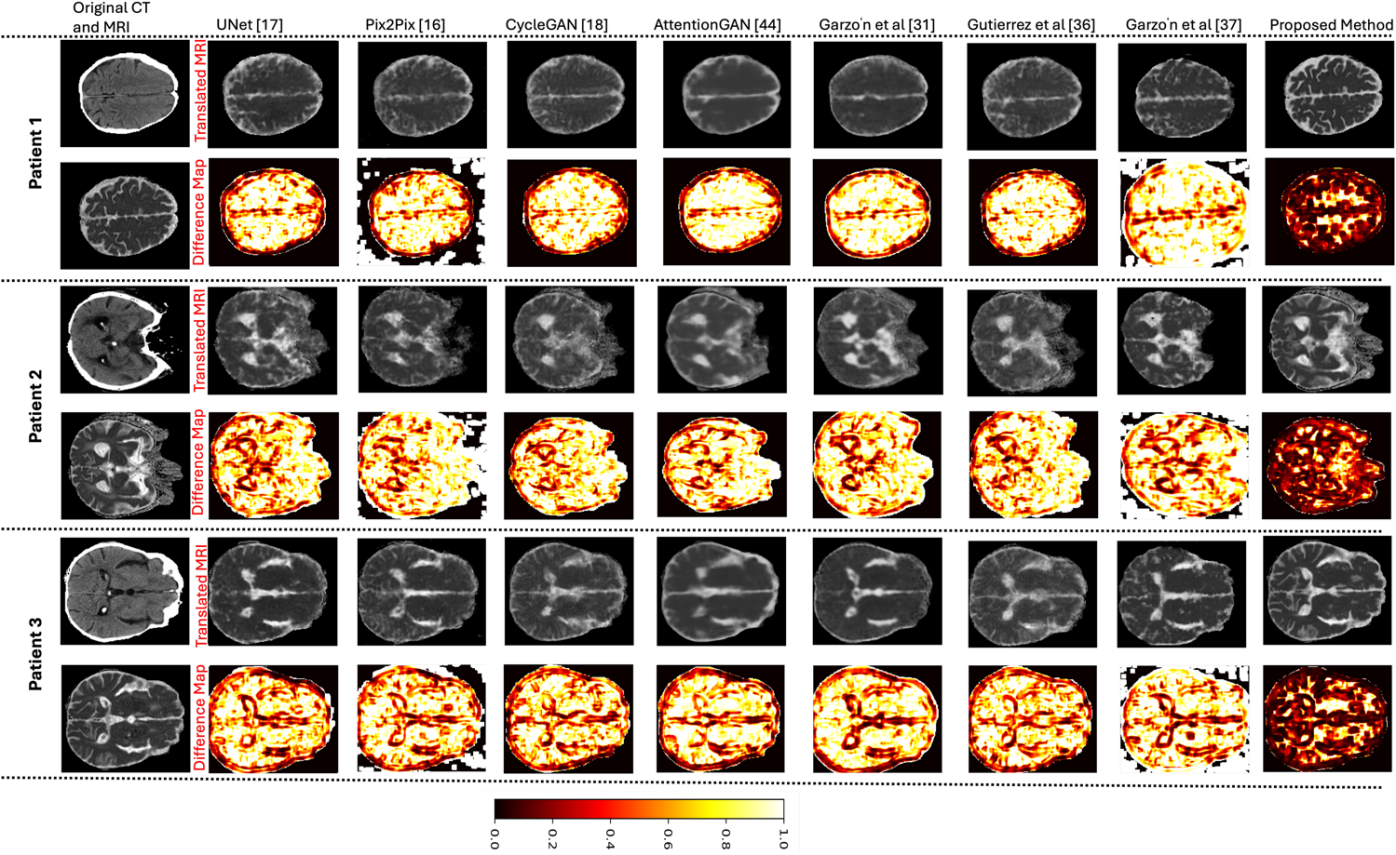
The qualitative evaluation results of the UNet [17], Pix2Pix [16], CycleGAN [18], AttentionGAN [43], Garzón et al. [31], Gutierrez et al. [36], Garzón et al. [37] and Proposed Method for the translation of CT images into ADC MRI images in three normal cases. Difference maps for each methods are displayed using a black-red-yellow colormap, where black = no difference (0) and yellow = complete difference (1)

**Fig. 5:**
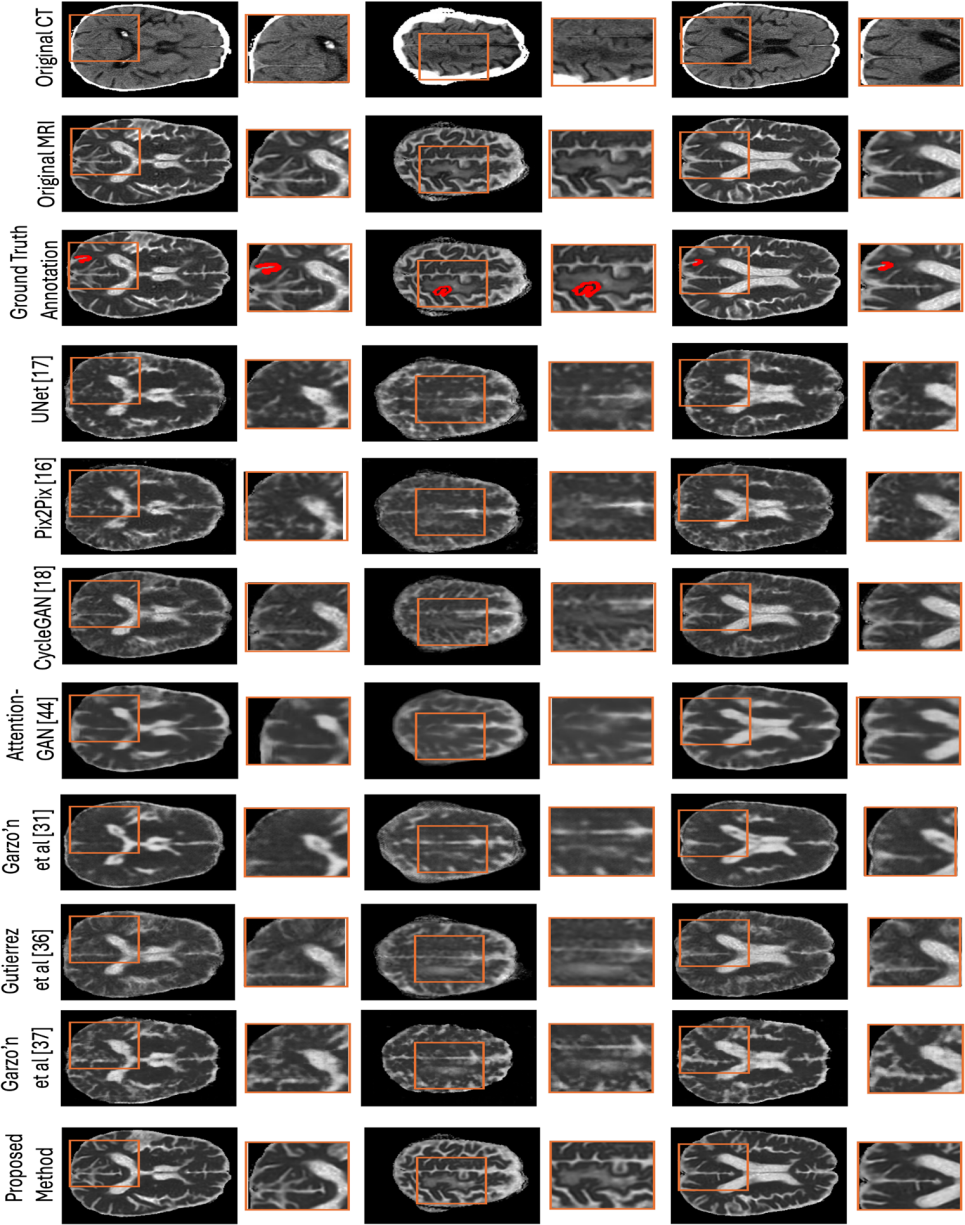
Some examples of original CT, original MRI, ground truth stroke lesion annotations, UNet [17], Pix2Pix [16], CycleGAN [18], AttentionGAN [43], Garzón et al. [31], Gutierrez et al. [36], Garzón et al. [37] and Proposed Method for the translation of CT images into ADC MRI images of stroke cases. The ground truth stroke lesion annotations drew red contours on the original ADC MRI images

Table 1 presents the detailed quantitative evaluation results of the proposed method with the benchmark methods for synthesising ADC MRI images from CT images. The experimental results demonstrate that the proposed method achieves better results than the benchmark methods in all five cases. The quantitative results also illustrate that the proposed method reduces the overall MSE in comparison to UNet [17], Pix2Pix [16], CycleGAN [18], AttentionGAN [43], Garzón et al. [31], Gutierrez et al. [36] and Garzón et al. [37] by 52.62$\%$, 58.67$\%$, 54.58$\%$, 56.15$\%$, 52.06$\%$, 54.34$\%$ and 75.97$\%$. Consequently, the proposed method increases the overall PSNR in contrast to UNet, Pix2Pix, CycleGAN, AttentionGAN, Garzón et al. [31], Gutierrez et al. and Garzón et al. [37] by 15.33$\%$, 18.22$\%$, 16.28$\%$, 16.92$\%$, 15.13$\%$, 11.01$\%$ and 31.91$\%$. Furthermore, the proposed method also increases the overall SSIM contrasted to UNet, Pix2Pix, CycleGAN, AttentionGAN, Garzón et al. [31], Gutierrez et al. and Garzón et al. [37] by 10.34$\%$, 10.34$\%$, 11.51$\%$, 9.52$\%$, 10.57$\%$, 11.39$\%$ and 20.75$\%$, respectively. To further evaluate the effectiveness of the proposed method, *P* values were computed using a Wilcoxon signed-rank two-sided test. The test results illustrate that the proposed method significantly outperforms the benchmark methods regarding MSE, PSNR and SSIM $(P < 0.01)$. Figure 4 shows examples of translated ADC MRI images of normal cases from UNet, Pix2Pix, CycleGAN, AttentionGAN, Garzón et al., Gutierrez et al., Garzón et al. and Proposed Method. Furthermore, the SSIM difference map was also calculated between the original ADC MRI images and the translated ADC MRI images. The SSIM difference map accounts for luminance, contrast and structure. The bright regions highlight areas where the images differ structurally, while the dark regions represent areas where the images are structurally similar. These qualitative results indicate that the UNet, Pix2Pix, CycleGAN, Garzón et al. [31] and Gutierrez et al. were not able to capture the structural similarity of the brain tissues fully. In contrast, AttentionGAN and Garzón et al. [37] generated smoothed translated ADC MRI images, which resulted in the loss of information. However, the proposed method maintains the shape well and generates translated ADC MRI images resembling ground-truth images. Figure 5 presents some original CT, original ADC MRI and translated ADC MRI images of stroke patients. The qualitative results for stroke cases demonstrate that the proposed method generates the translated ADC MRI images with more anatomical structure and low-signal regions associated with diffusion-restricted lesions.

### Ablation Study

An ablation study is performed to evaluate the effectiveness of the concatenation of brain mask information and fidelity-constrained loss in translating CT images into ADC MRI images. The ablation study consists of two experiments. The Wilcoxon signed-rank two-sided test was performed for both experiments to determine *P* values. In the first experiment, we compare the performance of traditional CycleGAN [18] with the performance of CycleGAN with brain mask information as a hard filter (multiplication) and a soft filter (concatenation). In the second experiment, the translation performance of the CycleGAN trained with the fidelity-constrained loss is compared with the traditional CycleGAN. Tables 2 and 3 present the quantitative evaluation results of the ablation study. The results illustrate that the concatenation of brain mask information in the CycleGAN achieves the best translation results $(P < 0.01)$. In addition, the results also demonstrate that utilising fidelity-constrained loss instead of pixel-based loss in the CycleGAN improves the translation performance as well $(P < 0.01)$.

**Table 2:** Quantitative evaluation results of Experiment 1 in the ablation study

Method	Number of	MSE	PSNR	SSIM (%)
	slices	(95% CI)	(95% CI)	(95% CI)
		*P* value	*P* value	*P* value
		434.70	21.93	79.37
CycleGAN [18]	122	(404.04, 465.37)	(21.64, 22.23)	(78.35, 80.39)
		4.86$\times$ $10^{-11}$	4.16$\times$ $10^{-11}$	3.56$\times$ $10^{-13}$
CycleGAN		632.68	20.15	75.67
w	122	(610.16, 691.00)	(19.87, 20.38)	(74.62, 76.57)
Mask Multiplication		3.56$\times$ $10^{-13}$	3.56$\times$ $10^{-13}$	3.56$\times$ $10^{-13}$
CycleGAN		262.75	24.24	86.15
w	122	(240.67, 284.85)	(23.84, 24.66)	(85.07, 87.23)
Mask Concatenation		–	–	–

**Table 3:** Quantitative evaluation results of Experiment 2 in the ablation study

Method	Number of	MSE	PSNR	SSIM (%)
	slices	(95% CI)	(95% CI)	(95% CI)
		*P* value	*P* value	*P* value
		434.70	21.93	79.37
CycleGAN [18]	122	(404.04, 465.37)	(21.64, 22.23)	(78.35, 80.39)
		1.90$\times$ $10^{-8}$	3.47$\times$ $10^{-8}$	6.27$\times$ $10^{-12}$
CycleGAN		373.86	22.61	81.23
w	122	(359.48, 417.04)	(22.12, 22.75)	(79.78, 81.86)
Fidelity-constrained Loss		–	–	–

### Representative ADC

**Fig. 6:**
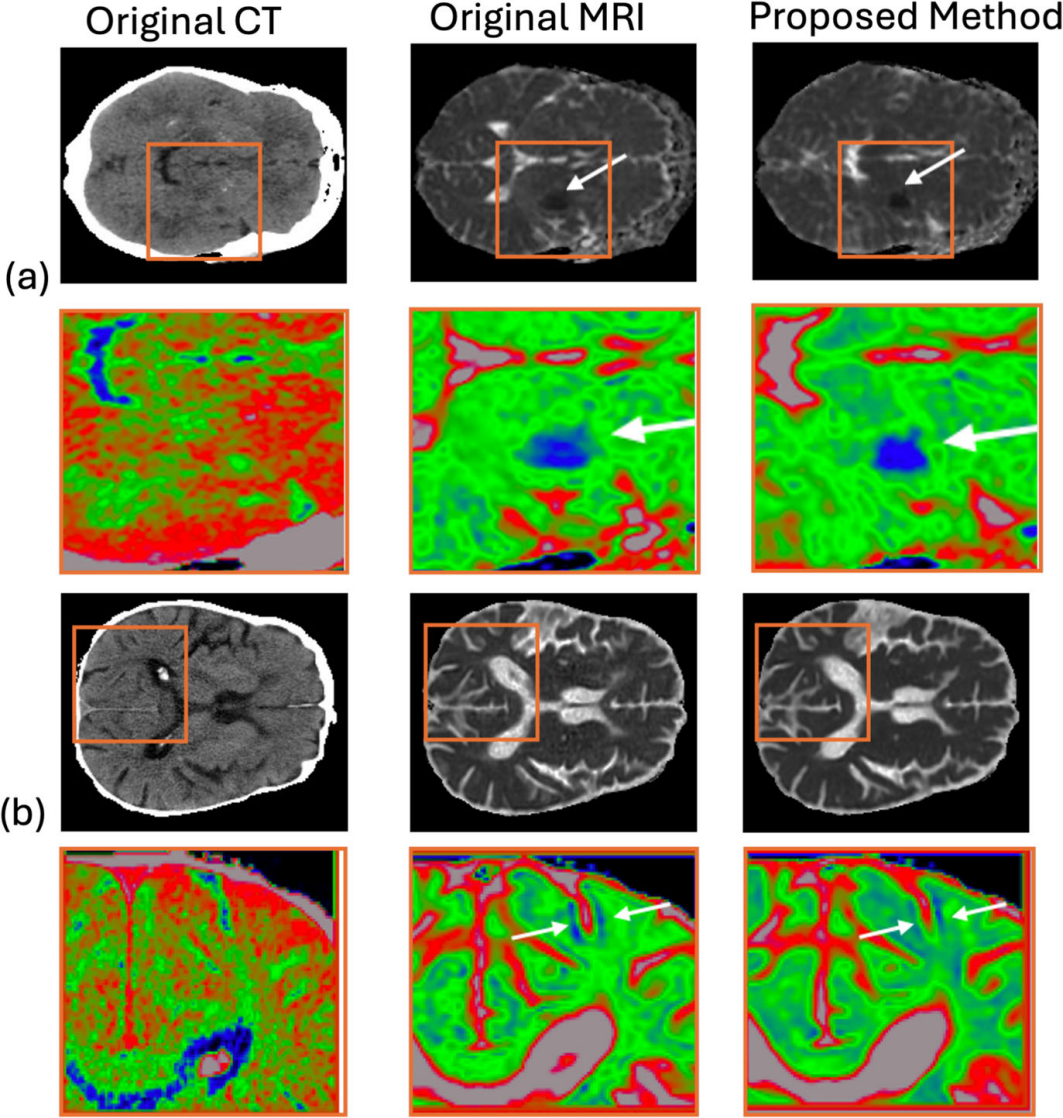
Example of low ADC signal lesions on MRI in **a** left basal ganglia and **b** cortex of two test cases with acute infarct lesions on original MRI and the proposed synthetic images derived from essentially normal CT. To improve contrast for visual examination, Rainbow colour maps (bottom row for each case) were generated using ImageJ, where blue represents low signal strength and red indicates high signal strength

The model outputs of the test cases were reviewed by a diagnostic radiologist with more than 20 years of experience in acute stroke imaging. Two representative test cases are shown in Fig. 6. Although no defined infarct was visible on the non-contrast CT, the proposed model was able to replicate potentially diagnostic replicates areas of low ADC signal in both deep nuclear and cortical infarcts.

## Discussion and Conclusion

In this work, a brain mask-guided and fidelity-constrained deep learning model was proposed to accurately translate CT images into ADC MRI images to aid in diagnosing stroke patients. The proposed method takes a brain mask as additional information with a CT image. The brain mask will be a soft guide for the model to focus on important regions for accurate translation. In addition, a fidelity-constrained loss is utilised instead of pixel-based losses. The fidelity-constrained loss focuses on maintaining the feature-level coherence compared to pixel-level similarity. Furthermore, the fidelity-constrained loss encourages the model to preserve global and local structural information by concentrating on semantic similarity and fine details.

The quantitative and qualitative evaluation results demonstrate that the proposed method achieves better global image performance than the benchmark methods for CT to MRI translation, including UNet [17], Pix2Pix [16], CycleGAN [18], AttentionGAN [43], Garzón et al. [31], Gutierrez et al. [36] and Garzón et al. [37]. Incorporating brain mask information and fidelity-constrained loss as a soft guide has significantly enhanced translation performance across several key metrics. UNet, Pix2Pix, Garzón et al. [31], Gutierrez et al., Garzón et al. [37] and CycleGAN use pixel-wise losses and enforce consistency between the translated images and real images without any anatomical guidance. As a consequence, they fail to emphasise the brain structures which are clinically relevant and usually learn mappings that do not align well with the anatomical fidelity. Furthermore, pixel-based losses might not be able to capture structural similarity effectively when dealing with modalities that have different intensity characteristics. This usually leads to smoothed translated images. AttentionGAN utilises dynamic attention masks to focus on clinically relevant regions. Furthermore, the dynamic nature of attention masks in AttentionGAN results in inconsistent supervision. Moreover, it also needs to learn the anatomical importance without domain priors, which can be challenging in CT images with low contrast. In comparison, the proposed method uses soft-guided brain masks, which allow the model to retain all the original information while encouraging focus on the clinically relevant brain regions.

The ablation study results illustrate that concatenating the brain mask (as a soft guide) outperforms its use as a hard filter (multiplying the brain mask). This could be because multiplying the brain mask limits the amount of information given to the model, reducing the diversity of the input features. Another reason could be the alteration of the intensity distribution of the input image with brain mask multiplication, which might affect the downstream feature extraction task. Furthermore, incorporating fidelity-constrained loss to preserve the fidelity of the translated image instead of pixel-wise losses achieves better results, demonstrating the effectiveness of fidelity-constrained loss. These results demonstrate the efficiency and efficacy of regional guidance and feature-level consistency for accurate translation in medical imaging.

Performance in global structural similarity measures may not infer clinical value in the detection of small focal lesions model or propagation of potential early CT correlates of the abnormal diffusivity that biologically underpins the ADC signal. This will rely on both sufficient changes being present in the CT image and the ability of the model to amplify these subtle contrast differences into the output image. Qualitatively, the proposed model shows the possibility of identifying diffusion-restricted lesions in both cortical and basal ganglia infarcts, but the segmentation of stroke lesions from the translated ADC MRI images is not explored in this study.

Our work has some limitations. Firstly, the dataset is limited due to the difficulties in acquiring paired CT and MRI datasets from stroke patients. The performance of the proposed method could be improved with the inclusion of more patient data. Furthermore, utilising a bigger and more diversified dataset may enhance the robustness and generalizability. In particular, different time durations of infarct will be necessary. The mismatch on MRI between diffusion and fluid attenuated inversion recovery (FLAIR) is a marker of acute (within 4.5 h) stroke, as ADC signal changes from cytotoxic redistribution may be visible within minutes, but oedema takes time for extracellular replenishment. Changes on CT may have more biological correlates with the oedema phase of MRI. However, acquiring such a large dataset is quite a challenging task. Future studies could also involve segmenting stroke lesions from synthetic MRI images to develop an automated framework for stroke diagnosis. Lastly, this paper emphasises the theoretical development, while detailed clinical assessment will be explored in future research.

In conclusion, this work demonstrates that the brain mask-guided and fidelity-constrained deep learning model generates synthetic ADC MRI images that closely resemble the original ADC MRI images and may have the potential to help clinicians in stroke diagnosis.

## Data Availability

The dataset is publicly available [46].

## Code Availability

The code will be made available upon reasonable request to the authors.
